# Comparison of automatic versus constant CPAP in elderly patients after major abdominal surgery: a randomized noninferiority trial

**DOI:** 10.1016/j.bjane.2025.844642

**Published:** 2025-05-17

**Authors:** Nguyen Dang Thu, Nguyen Thi Thuy, Le Sau Nguyen, Cong Quyet Thang, Nguyen Ngoc Thach, Nguyen Trung Kien

**Affiliations:** a Vietnam Military Medical University, Military Hospital 103, Department of Anesthesiology, Hanoi, Vietnam; b Friendship Hospital, Department of Anesthesiology, Hanoi, Vietnam; c Vietnam Military Medical Department, Hanoi, Vietnam

**Keywords:** Continuous positive airway pressure, Elderly, Laparotomy, Patient comfort, Pulmonary function tests

## Abstract

**Background:**

Geriatric patients undergoing major open abdominal surgery are at high risk for postoperative pulmonary complications and hypoxemia. Continuous Positive Airway Pressure (CPAP) after surgery may improve postoperative lung function. This randomized controlled trial compared two CPAP techniques ‒ automatic via nasal mask and constant via facial mask ‒ regarding pulmonary function and patient tolerance.

**Methods:**

Sixty patients (≥ 60 years) were randomized (1:1) to receive either automatic CPAP (2–10 cm H_2_O) via a nasal mask (Group A) or constant CPAP (7.5 cm H_2_O) via a facial mask (Group C) upon arrival in the post-anesthesia care unit. Oxygenation (PaO_2_, PaO₂/FiO₂, SpO_2_) and spirometry (FVC, FEV_1_, PEF) were assessed preoperatively, postoperatively, and one hour after treatment. Comfort scores (0–10, with 0 indicating the best comfort) and complications were recorded.

**Results:**

PaO₂/FiO_2_ improvement was lower in Group A (32.6 ± 26.3 mmHg) than in Group C (52.9 ± 40.1 mmHg; p = 0.023). FVC improvement was also lower in Group A (3.7% ± 4.0%) than in Group C (6.7% ± 4.9%; p = 0.012). However, Group A had better tolerance, with lower comfort scores (2 [2–3] vs. 3 [2–4], p = 0.002). Pulmonary function benefits were more pronounced in patients over 70 and those undergoing upper abdominal surgery.

**Conclusion:**

Both CPAP techniques prevent pulmonary decline in geriatric patients post-surgery. While automatic CPAP provides better comfort, constant CPAP improves oxygenation. Although our findings are short-term, they suggest that CPAP mode selection should be tailored based on patient-specific needs.

## Introduction

Postoperative pulmonary complications frequently arise following major abdominal surgery, significantly contributing to higher morbidity, extended hospitalization, and elevated mortality rates.^1^^,^^2^ Hypoxemia and respiratory alterations reach their peak in the initial hours post-surgery, potentially leading to acute respiratory failure ‒ an occurrence observed in 30% to 50% of individuals after upper abdominal surgery.^3^ This issue is particularly pronounced in the aging population, where surgical volume is rising.^4^ The deleterious physiological changes associated with aging, coupled with the damaging effects of comorbidities on the pulmonary system, result in a heightened risk of perioperative pulmonary outcomes among geriatric patients.^3^^,^^5^ The incidence rates of postoperative pulmonary complications in geriatric patients range from 1% to 23%, depending on surgical factors.^6^ This underscores the importance of timely and effective treatment during the early postoperative period to prevent hypoxemia and its associated complications in these populations.

Postoperative Continuous Positive Airway Pressure (CPAP) application may effectively reduce the risk of pulmonary complications in both preventive and therapeutic settings.^7^, ^8^, ^9^ CPAP involves utilizing a high-pressure gas source or machine to deliver positive pressure during both inspiration and expiration. This pressure can be consistently delivered or varied through a nasal airway or face mask.^10^ Constant CPAP via a face mask may be poorly tolerated, especially over extended periods, and requires frequent staff adjustments to prevent air leakage, particularly in elderly patients with age-related facial changes.^11^ Nasal automatic CPAP devices, providing positive pressure in response to abnormalities in the breathing pattern, have proven effective, well-tolerated, and widely applied for sleep apnea-hypopnea syndrome patients.^12^ This highlights the potential benefits of automatic CPAP for postoperative care. However, while most CPAP studies focus on pulmonary function improvement, fewer address discomfort during treatment. Additionally, although CPAP is widely used postoperatively, limited evidence exists comparing automatic and constant CPAP in geriatric patients, particularly regarding oxygenation and patient tolerance.

This study assesses the impact of nasal automatic CPAP compared to face mask constant CPAP on postoperative oxygenation, respiratory mechanics, and comfort in geriatric patients undergoing major open abdominal surgery.

## Method

This single-center randomized controlled trial, approved by the Vietnam Military Medical University Ethics Committee (n° 3977/QĐ-HVQY) and registered on ClinicalTrials.gov (NCT06260826), followed the Declaration of Helsinki and CONSORT guidelines. All patients provided informed consent.

### Patient population

We enrolled 60 patients over 60 years old undergoing major open abdominal surgery (e.g., gastrectomy, colectomy, proctocolectomy, hepatectomy) from December 2021 to August 2022. Exclusion criteria included preoperative non-invasive ventilation, airway deformities, bullous emphysema, suspected bronchopleural fistula, facial abnormalities, delayed extubation (> 4 hours), non-epidural anesthesia, or inability to consent. Additionally, we excluded patients with suspected sleep apnea syndrome based on clinical symptoms and risk factors, such as obesity (BMI > 30), loud snoring, and witnessed apneas during sleep.

### Anesthesia protocol

Patients received standardized anesthetic management per hospital protocols. Before induction, an epidural catheter was placed in the epidural space at D7–9 for upper abdominal surgery and at L1‒3 for lower abdominal surgery. A 0.2% bupivacaine solution was initially administered as a 5 mL bolus and maintained at 5 mL.h^-1^ during surgery. General anesthesia was induced with propofol (1–2 mg.kg^-1^), fentanyl (1–2 µg.kg^-1^), and rocuronium (0.6–0.8 mg.kg^-1^), followed by intubation after 3 minutes of manual ventilation. Anesthesia was maintained with sevoflurane (0.5–0.8 MAC) and oxygen/air (FiO_2_ = 0.4). Rocuronium was given based on TOF (train-of-four) monitoring. Fentanyl and epidural rates were adjusted to maintain the Surgical Pleth Index at 40–70. Intraoperatively, fluid balance was managed with Ringer’s lactate and colloid solutions, based on urine output, blood loss, and central venous pressure.

The ventilator was set in Pressure-Controlled Volume Guarantee (PCV-VG) mode with tidal volume 6–8 mL.kg^−1^ (ideal body weight), inspiration: expiration ratio of 1:2, positive end-expiratory pressure of 5 cm H_2_O, and the respiratory rate (9–12 per minute) was adjusted to maintain EtCO_2_ between 35–40 mmHg. Alveolar recruitment maneuvers were performed every 30–45 minutes. The patient was extubated when fully awake, spontaneously breathing, and TOF > 90%. Nausea and vomiting were prophylactically managed with dexamethasone (4 mg) and ondansetron (4 mg). Postoperative pain was managed with continuous epidural analgesia (bupivacaine 0.125% + fentanyl 2 µg.mL^-1^), paracetamol (15 mg.kg^-1^), and nefopam (20 mg).

### Randomization and interventions

After extubation, all patients were positioned with a 30° upper body elevation. CPAP treatment began as soon as patients could cough and clear phlegm. Randomization into Group A or Group C (1:1 ratio) was performed using a computer-generated list, with allocation concealed in numbered, sealed, opaque envelopes opened by a research nurse upon the patient's arrival at the PACU.

Group A: automatic CPAP (JPAP system, Metran, Japan) delivered via a nasal mask with a reference pressure of 7 cm H_2_O during a 5-minute ramp time (in 0.5 cm H_2_O increments). After that, the pressure was allowed to vary within a 2–10 cm H_2_O range during treatment, with O_2_ at 6 L.min^-1^.

Group C: constant CPAP (O2-Max Trio, Pulmodyne, USA) delivered via a facial mask with a pressure set at 7.5 cm H_2_O and FiO_2_ at 30%, both fixed throughout the treatment.

Patients received 1 hour of CPAP therapy. Those unable to tolerate it were treated according to standard PACU protocols and excluded from further analysis. PACU discharge was determined using a modified Aldrete score.^13^

### Outcome measures

The primary outcome was the PaO_2_/FiO_2_ ratio. Arterial blood gas was measured at three time points: before surgery, at fixed postoperative intervals ‒ including upon arrival at the PACU ‒ and immediately after CPAP treatment using the Cobas B221 blood gas machine (Roche, Basel, Switzerland) for all patients.

Secondary outcome measures included spirometry parameters (forced vital capacity-FVC, forced expiratory volume in the first second-FEV1, FEV1/FVC ratio, and peak expiratory flow-PEF), assessed using Spirobank II Advanced (Medical International Research, Roma, Italia) in a 45° upper body elevation position, concurrently with blood gas assessment. After CPAP treatment, patients rated their overall comfort on a numeric rating scale (NRS, 0–10, where 0 indicated the best comfort and 10 the worst), and other complications related to CPAP were recorded.

### Sample size and statistical analysis

According to previous studies that have reported oxygenation improvements (ΔPaO_2_) of 15 mmHg (SD = 18 mmHg) and a reduction in postoperative respiratory failure with CPAP or non-invasive ventilation compared to traditional oxygen therapy in morbidly obese postoperative patients,^14^, ^15^, ^16^ the sample size was calculated to be 48 (24 per group) using an alpha of 0.05 and a power of 0.8. After accounting for a 20% dropout rate, 60 patients were included, with 30 in each group.

Data are presented as mean ± SD and range for continuous variables or as numbers and percentages for categorical variables. Variable distribution was assessed using histograms and the Kolmogorov-Smirnov test. Group differences were analyzed using Student’s *t*-test or Mann-Whitney *U* test. Within-group variations for continuous variables were assessed using two-way repeated measure ANOVA with Tukey’s post hoc tests. Categorical variables were analyzed using Chi-Squared or Fisher’s exact tests. IBM SPSS Statistics, version 19.0 (IBM Corp., Armonk, N.Y., USA), was used for statistical analyses. A p-value < 0.05 was considered statistically significant.

## Results

**Figure 1:**
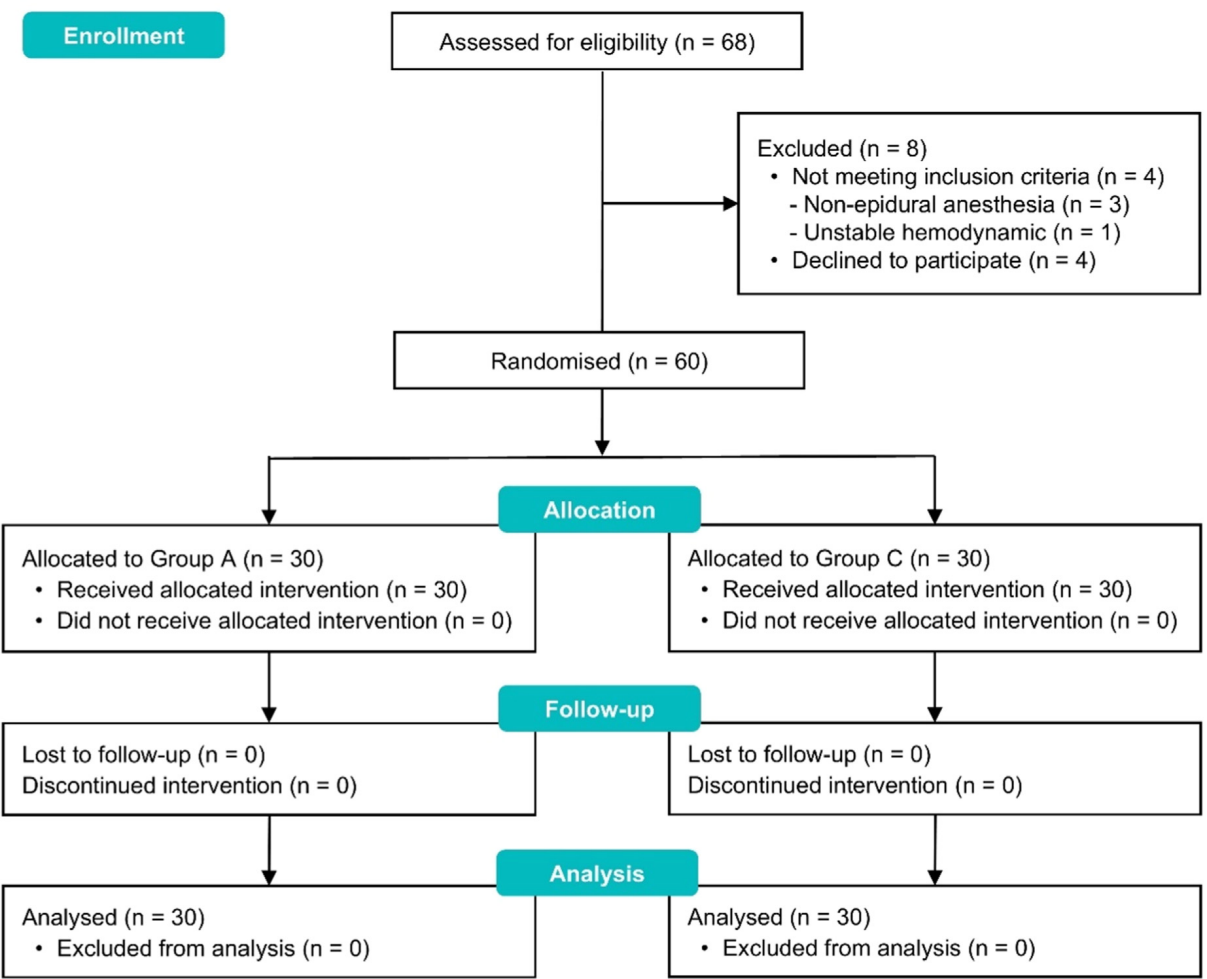
CONSORT diagram.

After randomization, 30 patients were included in each group, with no cases excluded due to CPAP intolerance or incomplete follow-up, as presented in the CONSORT-compliant flow diagram (Fig. 1). There were no significant differences between the groups regarding preoperative characteristics, surgery, and anesthesia features (Table 1).

**Table 1:** Demographic data.

	Group A (n = 30)	Group C (n = 30)	p-value
**Age (yr)**	73.1 ± 8.3 [62–88]	73.7 ± 7.0 [60–86]	0.775
**Weight (kg)**	59.0 ± 8.7 [42–80]	58.8 ± 7.9 [42–75]	0.926
**Height (cm)**	163.2 ± 6.5 [150–175]	161.7 ± 5.3 [150–171]	0.366
**BMI**	22.1 ± 2.6 [16.8–27]	22.4 ± 2.3 [17.8–26.9]	0.637
**Gender (Male/Female)**	25/5	24/6	0.739
**ASA grade (II/III)**	10 /20	11/19	0.787
**Preoperative respiratory disease**			
Asthma, n (%)	0	1 (3.3)	1
COPD, n (%)	1 (3.3)	0	1
Smoker, n (%)	9 (30)	9 (30)	1
**Surgical site**			1
Upper abdominal, n (%)	22 (73.3)	22 (73.3)	
Lower abdominal, n (%)	8 (26.7)	8 (26.7)	
Surgery length (min)	178 ± 50 [90–315]	162 ± 60 [80–310]	0.253
Anesthesia length (min)	209 ± 49 [115–340]	187 ± 59 [110–345]	0.129
**Anesthetic agents**			
Fentanyl (µg)	100	100	1
Rocuronium (mg)	55.2 ± 17.2 [40–110]	51.7 ± 10.2 [40–110]	0.343
Sevoflurane (mL)	53.0 ± 13.0 [27–85]	46.6 ± 14.1 [23–78]	0.071

Data are expressed as mean ± SD and [range]; BMI, Body Mass Index; ASA, American Society of Anesthesiology, COPD, Chronic Obstructive Pulmonary Disease. Student’s *t*-test, Chi-Squared test, or Fisher’s exact test.

**Figure 2:**
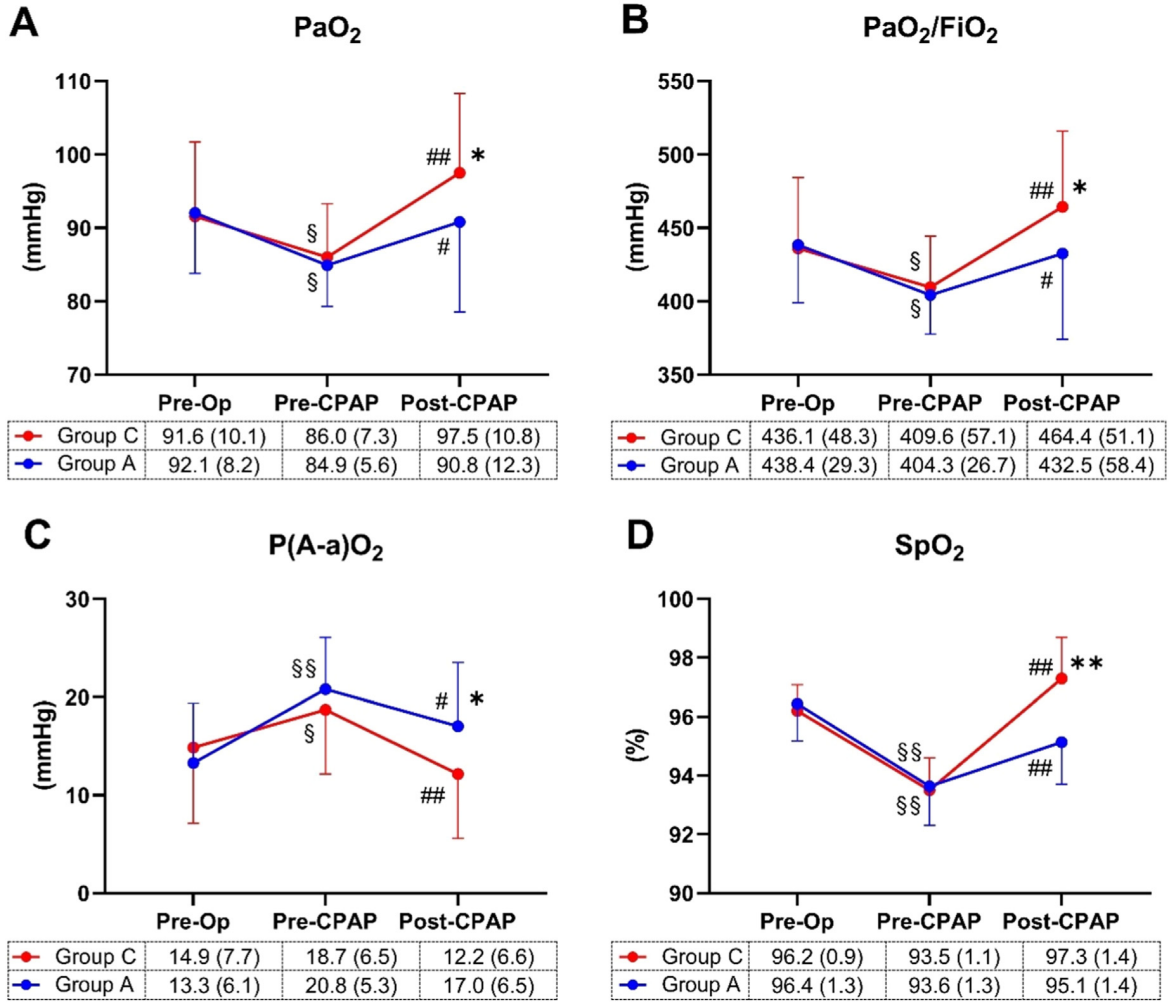
Blood gas analyses and pulse oximetry values. The arterial partial pressure of oxygen (PaO_2_; A), the ratio of PaO_2_ to the fraction of inspired oxygen (PaO_2_/FiO_2_; B), the alveolar-arterial gradient (P(A-a)O_2_; C) and the saturation of peripheral oxygen (SpO_2_; D) were recorded before anesthesia (Pre-Op), after anesthesia before applying CPAP (Pre-CPAP) and after CPAP (Post-CPAP). All measurements were taken after 5 minutes of breathing room air. Data are expressed as means with SD. Differences were estimated by two-way repeated-measures ANOVA with Tukey’s post-hoc test (*p < 0.05, **p < 0.001, between groups; ^§^p < 0.05, ^§§^p < 0.001, within the group between Pre-CPAP and Pre-Op; ^#^p < 0.05, ^##^p < 0.001; within the group between Post-CPAP and Pre-CPAP).

Figure 2 illustrates the oxygenation parameters. Post-surgery, PaO_2_, PaO_2_/FiO_2_, and SpO_2_ values before CPAP (pre-CPAP) significantly decreased in all patients compared to pre-surgery (pre-op), with no differences between groups. Following CPAP treatment (post-CPAP), PaO_2_ (Fig. 2A) improved in both groups (adjusted p = 0.011 in Group A, < 0.001 in Group C; within-group comparison), showing an interaction between group and time [F(2, 58) = 3.67, p = 0.031]. PaO_2_ in Group C was higher than in group A (p = 0.004, between groups). PaO_2_/FiO_2_ values (Fig. 2B) exhibited a pattern similar to PaO_2_.

After surgery, the alveolar-arterial gradient (P(A-a)O_2_, Fig. 2C) significantly increased in both Groups A and C (adjusted p < 0.001 and 0.012, respectively). CPAP treatment effectively reversed this increase, with adjusted p-values of 0.014 (Group A) and < 0.001 (Group C). A significant group and time interaction was observed [F(2, 58) = 6.11, p = 0.004], and Group C showed a significantly lower P(A-a)O_2_ than Group A (p = 0.001, between groups).

The pulse oximetry values (SpO_2_) displayed a similar pattern to the PaO_2_ parameter (Fig. 2D).

**Figure 3:**
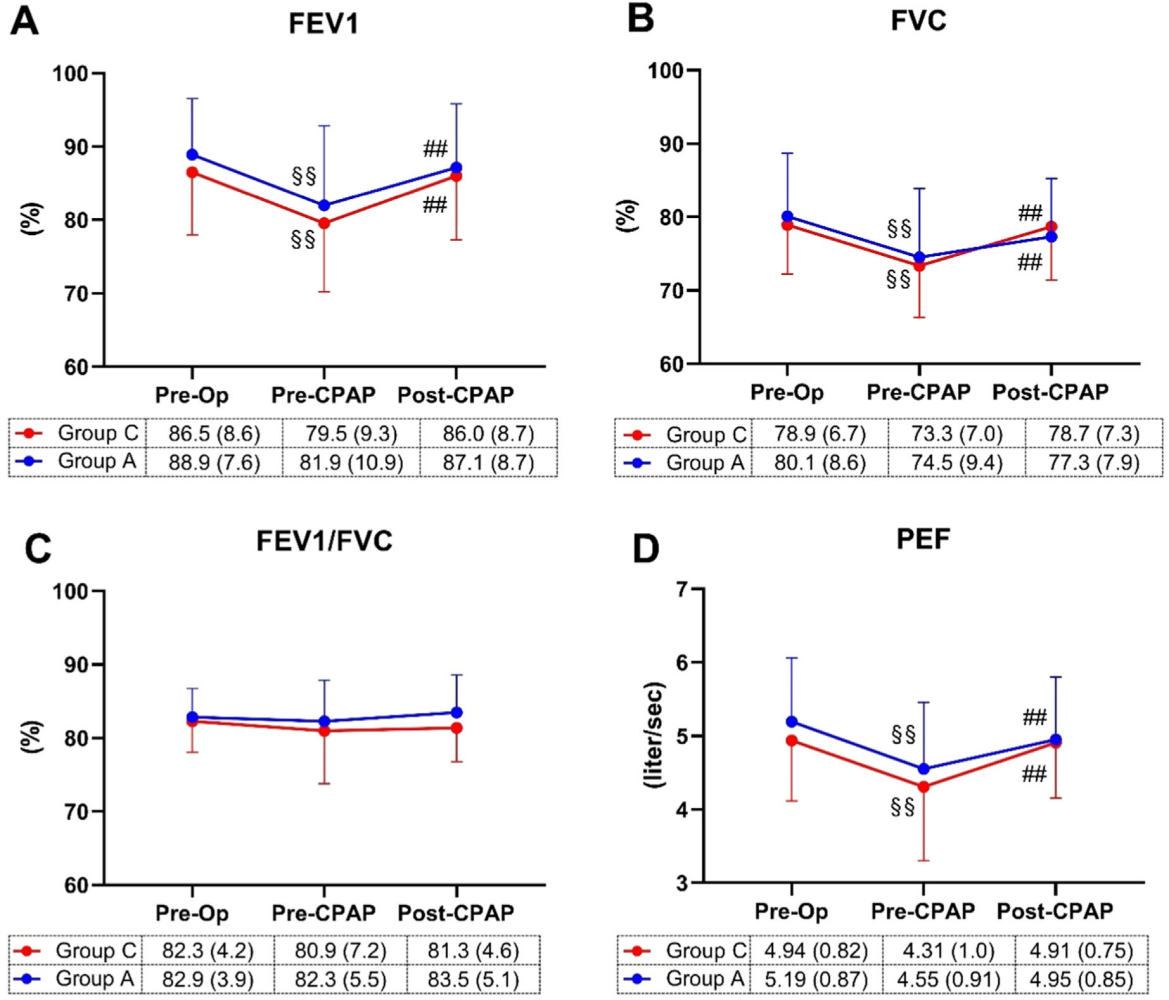
Respiratory Mechanics. The Forced Expiratory Volume in the first second (FEV1; A), Forced Vital Capacity (FVC; B), FEV1/FVC (C), and Peak Expiratory Flow (PEF; D) values were recorded before Anesthesia (Pre-Op), after anesthesia before applying CPAP (Pre-CPAP) and after CPAP (Post-CPAP). Data are expressed as means with SD. Differences were estimated by two-way repeated-measures ANOVA with Tukey’s post-hoc test (^§^p < 0.05, ^§§^p < 0.001, within the group between Pre-CPAP and Pre-Op; ^#^p < 0.05, ^##^p < 0.001; within the group between Post-CPAP and Pre-CPAP).

Figure 3 illustrates respiratory mechanics parameters. Postoperative FEV1 (Fig. 3A), FVC (Fig. 3B), and PEF (Fig. 3D) values decreased compared to preoperative levels in both groups, with improvement following CPAP application. No significant group differences were observed in FEV1, FEV1/FVC (Fig. 3C), and PEF at all measurement time points. However, there was an interaction between group and time in FVC values [F(2, 58) = 6.02, p = 0.004].

**Figure 4:**
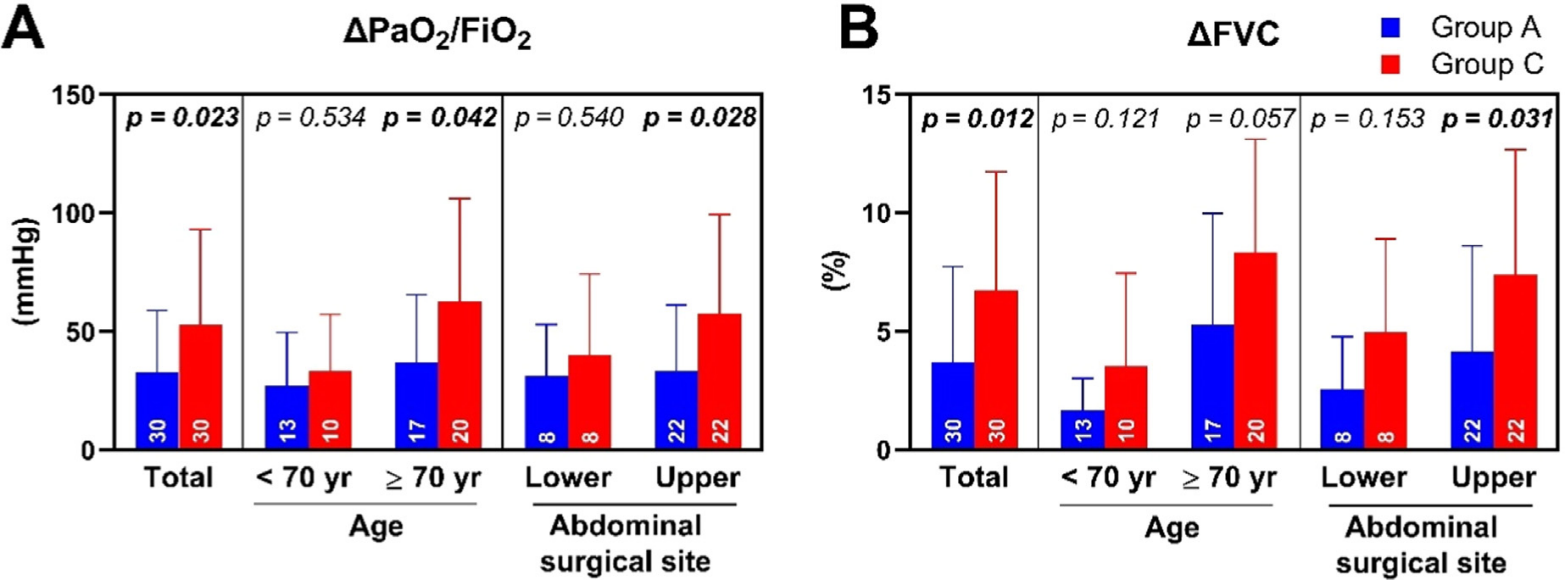
The change in lung function after applying CPAP and subgroup. The change induced by applying CPAP (Post-CPAP – Pre-CPAP) in the ratio of the Arterial Partial pressure of Oxygen to the Fraction of Inspired Oxygen (ΔPaO_2_/FiO_2_; A) and Forced Vital Capacity (ΔFVC; B) were subgrouped by age and abdominal surgical site. Data are expressed as means with SD. Differences were estimated by Student’s *t*-test. Numbers inside the bars indicate the number of participants.

After CPAP treatment, the improvement in PaO_2_/FiO_2_ (ΔPaO_2_/FiO_2_ = post-CPAP PaO_2_/FiO_2_ value − pre-CPAP PaO_2_/FiO_2_ value) in Group A (32.6 ± 26.3 mmHg) was lower than in Group C (52.9 ± 40.1 mmHg) with p = 0.023 (Fig. 4A). Similarly, the improvement in FVC (ΔFVC = post-CPAP FVC value − pre-CPAP FVC value) in Group A (3.7 ± 4.0%) was lower than in Group C (6.7 ± 4.9%) with p = 0.012 (Fig. 4B). Notably, the difference between CPAP treatment groups in ΔPaO_2_/FiO_2_ (mmHg) was more pronounced in patients aged ≥ 70 (95% CI: 2.88–53.30) than those < 70 (95% CI: -16.70 to 23.45) and in upper (95% CI: 2.79–46.12) vs. lower abdominal surgery (95% CI: -21.79 to 39.79), despite similar intraoperative ventilation settings and opioid use across subgroups. A similar trend was observed in ΔFVC (%) for upper (95% CI: 0.30–6.25) vs. lower abdominal surgery (95% CI: -1.01 to 5.82).

Postoperative complications and comfort data are detailed in Table 2. While some Group A patients reported device noise as a concern, discomfort in Group C was more commonly associated with pressure, mouth dryness, and mask contact with the face. Overall, patients treated with automatic CPAP reported greater comfort than those receiving constant CPAP.

**Table 2:** Patient-reported comfort and complications.

	Group A (n = 30)	Group C (n = 30)	p-value
**Comfort Score, median (IQR)**	2 (2–3)	3 (2–4)	**0.002**
**Uncomfortable due to**			
Noise, n (%)	5 (16.7)	1 (3.3)	0.194
Pressure, n (%)	1 (3.3)	9 (30)	**0.012**
Dryness of mouth, n (%)	1 (3.3)	5 (16.7)	0.194
**Facial erythema**	0	3 (10)	0.237
**Length of PACU stay, h**	5.4 ± 1.2	5.6 ± 1.3	0.765
**Hospital mortality, n (%)**	1 (3.3)	1 (3.3)	1

Numeric Rating Scale (NRS), 0–10: with the number 0 indicating the best possible comfort and 10 the worst.

Mann-Whitney *U* test, Fisher’s exact test, Student’s *t*-test.

No patient experienced gastric distension, conjunctival congestion, pneumothorax, or hypotension due to CPAP application during PACU time, and the length of PACU stay was similar between groups. Additionally, no patients in either group developed severe pulmonary complications requiring reintubation or ICU readmission for respiratory failure within 48 hours postoperatively. The hospital mortality rate was similar between groups.

## Discussion

Our study shows that both automatic and constant CPAP techniques improve gas exchange and respiratory mechanics in geriatric patients undergoing major open abdominal surgery. Constant CPAP leads to greater improvements in oxygenation and FVC compared to automatic CPAP, while the latter provides higher comfort scores during treatment.

CPAP treatment, whether automatic or constant, improved postoperative oxygenation in geriatric patients. The observed decline in postoperative pulmonary function can be attributed to hypoventilation,^3^ atelectasis,^17^ and an increased alveolar-arterial gradient.^18^ Here, we found that after 1 hour of CPAP, PaO_2_/FiO_2_ and FVC improved by 52.9 mmHg and 6.7% with the constant technique and 32.6 mmHg and 3.7% with the automatic technique. Our findings with constant CPAP are consistent with previous studies after major abdominal surgery, though data specifically on elderly patients remain limited. Hatice Yağlıoğlu et al. reported a 137-mmHg increase in PaO₂/FiO₂ and a 14.6% rise in expiratory tidal volume in patients (mean age 60–61 years) with COPD comorbidities, which may explain their greater improvement.^19^ Similarly, a 32 mmHg increase was observed in younger, morbidly obese patients (mean age 42.6 years),^20^ whereas in older patients (mean age 67–68 years) after major abdominal surgery, PaO₂/FiO₂ improved by only 10 mmHg despite 6 hours of CPAP.^21^ This may be due to intermittent mask CPAP rather than continuous application.^21^ The lung expansion effects of CPAP ‒ preventing airway collapse, promoting alveolar recruitment, and reducing the work of breathing ‒ likely contributed to the observed improvements in FEV₁, FVC, and PEF with both automatic and constant CPAP in our study. A similar 5.7% increase in FVC was reported by Joana Guimarães et al. with constant CPAP.^22^ However, some studies have found no significant improvement in FVC and FEV_1_ with CPAP compared to conventional oxygen therapy postoperatively.^20^^,^^22^^,^^23^ These discrepancies may be due to differences in treatment duration^23^ or patient characteristics.^20^^,^^22^ To date, the effect of postoperative automatic CPAP on pulmonary function has not been published. Our findings suggest that the pulmonary function benefits of CPAP vary depending on patient characteristics.

The observed differences in oxygenation and lung function improvement between the automatic CPAP and constant CPAP groups may be attributed to variations in the delivered airway pressure, which play a primary role in lung expansion. The lower pressure may not be sufficient to open micro-atelectasis areas.^24^ A study on sleep apnea-hypopnea syndrome patients found that patients using automatic CPAP slept at a mean pressure lower than those using constant CPAP.^25^^,^^26^ While the airway pressure is maintained with the constant CPAP, the effective pressure with automatic CPAP can vary within a given subject in each breath cycle, depending on body position, fatigue level, sedative stage, and upper airway characteristics.^26^ Alveolar pressure created by CPAP can vary across the respiratory cycle among different patients. Our population study is mostly within the normal or lower range of BMI, Mallapati levels 1 and 2, and lying in a head-up position resulting in the pressure delivered in Group A possibly being lower than the pressure in Group C. Additionally, patients in Group A used nasal masks, which can lead to air leakage through the mouth, further contributing to differences in effective pressure between the groups.

Although constant CPAP resulted in greater pulmonary function improvements than automatic CPAP, we found that automatic CPAP treatment with a nasal mask provided patients with higher comfort scores than constant CPAP with a face mask. Moreover, no patients required a break or exhibited non-adherence during the 1-hour treatment in either group. Various studies have evaluated patient tolerance when comparing automatic and constant CPAP for the treatment of obstructive sleep apnea, consistently reporting a preference for automatic CPAP.^27^ Poor compliance with face mask constant CPAP therapy is a well-recognized issue, particularly in long-term treatment, with non-adherence rates ranging from 46% to 83%.^28^ Our study is the first to evaluate patient comfort during automatic versus constant CPAP treatment in the acute postoperative setting. Discomfort with constant CPAP was more commonly associated with pressure and mask contact with the face. In a study by Jens T. F. Osterkamp et al.,^21^ the overall comfort score had a median (IQR) of 2 (1–3), with 27% of patients reporting discomfort due to pressure ‒ similar to our findings. However, skin trauma or facial erythema was not reported in their study, possibly because the face mask was applied intermittently. The pressure variation in automatic CPAP devices provides greater comfort for patients compared to constant pressure. Additionally, using a nasal mask can reduce mouth dryness and benefit patients with mask fit issues, postoperative delirium, anxiety, or claustrophobia. Enhanced comfort with nasal automatic CPAP may support prolonged treatment duration, contributing to hemodynamic stability and improved lung function.

Notably, the more significant benefit of both CPAP techniques on lung function improvement was found in patients aged over 70 and/or those undergoing upper abdominal surgery. Age, identified as an independent risk factor for postoperative pulmonary complications, exhibits an increasing odds ratio (95% CI) of 2.1 (1.7–2.6) for individuals aged 60 to 69 compared to those under 60, with the risk further escalating with advanced age.^18^ This susceptibility is attributed to their limited physiological reserve, age-related frailty, higher airway closing capacity, and lower ventilation-perfusion ratios.^29^ Furthermore, the diaphragm undergoes more cephalad displacement and splinting during upper abdominal surgery, combined with limited respiratory excursion induced by pain, exacerbating the reduction in functional residual capacity^30^ and postoperative hypoxemia. High-risk patients, with advanced age as a contributing factor, also derived more benefits from CPAP treatment after lung resection surgery.^31^ Therefore, we assert that the prophylactic use of CPAP holds greater clinical relevance for advanced-age patients and/or those undergoing upper abdominal surgery.

Our study has some limitations. We applied CPAP for one-hour post-extubation and evaluated its short-term effects on pulmonary function and patient tolerance in the PACU. Longer CPAP applications and more detailed assessments of pulmonary complications using lung imaging could provide stronger evidence regarding the benefits and risks of these techniques in postoperative settings. Among the excluded subjects, some may have had poor CPAP tolerance, potentially biasing the overall patient tolerance results. Additionally, our study population primarily consisted of elderly patients, which may limit its generalizability. Further research on individuals with specific comorbidities, particularly pre-existing lung diseases, could provide valuable insights into these unique populations. Moreover, a larger sample size would strengthen the reliability of our findings and allow for more robust subgroup analyses.

Nevertheless, our study provides initial evidence to guide anesthesiologists in selecting a CPAP technique for postoperative patients, balancing lung function improvement with patient comfort. While constant CPAP offers superior gas exchange benefits, making it ideal for patients at high risk of immediate postoperative hypoxemia and reduced functional residual capacity, automatic CPAP may be preferable for those prioritizing comfort with lower risks, and longer CPAP application may further enhance its benefits.

## Conclusion

Both automatic and constant CPAP techniques enhance respiratory function, including gas exchange and mechanical respiration, in elderly patients undergoing major open abdominal surgery, with particularly notable benefits in advanced age and upper abdominal surgery patients. While both CPAP techniques effectively improve postoperative oxygenation, automatic CPAP may be preferable for patients prioritizing comfort, whereas constant CPAP provides superior gas exchange improvements. Further research is needed to determine optimal duration and patient selection criteria.

## Availability of data and materials

Data for this study are available from the corresponding author upon reasonable request.

## Authors’ contributions

Concept and study design: Nguyen Thi Thuy, Nguyen Dang Thu, Le Sau Nguyen, Cong Quyet Thang, Nguyen Ngoc Thach.

Data acquisition: Nguyen Thi Thuy, Le Sau Nguyen.

Data analysis and interpretation: Nguyen Dang Thu, Nguyen Thi Thuy.

Writing-original draft: Nguyen Dang Thu, Nguyen Thi Thuy.

Writing, review, and editing: Nguyen Dang Thu, Nguyen Thi Thuy, Le Sau Nguyen, Cong Quyet Thang, Nguyen Ngoc Thach, Nguyen Trung Kien.

All authors are anaesthesiologists and have read and approved the final manuscript.

## Funding

The author(s) received no financial support for the research, authorship, and/or publication of this article.

Clinical Trial.gov identified NCT06260826 (15/02/2024)

https://clinicaltrials.gov/study/NCT06260826

## Conflicts of interest

The authors declare no conflicts of interest.
